# Efficacy of Neoadjuvant Chemotherapy with Capecitabine plus Oxaliplatin in the Treatment of Locally Advanced Sigmoid Colon Cancer Invading the Urinary Bladder: A Report of Three Cases

**DOI:** 10.1155/2019/8129358

**Published:** 2019-03-27

**Authors:** Tsutomu Takenami, Shingo Tsujinaka, Jun Takahashi, Sawako Tamaki, Ryo Maemoto, Rintaro Fukuda, Hideki Ishikawa, Nao Kakizawa, Fumi Hasegawa, Rina Kikugawa, Yasuyuki Miyakura, Koichi Suzuki, Akira Tanaka, Toshiki Rikiyama

**Affiliations:** ^1^Department of Surgery, Saitama Medical Center, Jichi Medical University, Japan; ^2^Department of Pathology, Saitama Medical Center, Jichi Medical University, Japan

## Abstract

**Introduction:**

We herein present three cases of locally advanced colon cancer (LACC) invading the urinary bladder, in whom combined neoadjuvant chemotherapy with surgical intervention was effective in disease control and preserving urinary function.

**Case Presentation:**

Before neoadjuvant chemotherapy, all three cases underwent loop transverse colostomy for symptomatic colonic obstruction. Case 1: after 6 courses of capecitabine plus oxaliplatin (CAPOX), we performed sigmoid colectomy and partial resection of the bladder. The histological examination revealed pathological complete response (pCR). The final diagnosis was ypStage 0 (ypT0ypN0M0). Case 2: after 13 courses of CAPOX plus bevacizumab, we performed Hartmann's operation with partial resection of the bladder. The histological examination revealed pCR. The final diagnosis was ypStage 0 (ypT0ypN0M0). Case 3: after 6 courses of chemotherapy with CAPOX plus bevacizumab, we performed sigmoid colectomy and partial resection of the bladder. The pathological response was grade 1a according to the Japanese Classification of Colorectal Carcinoma. The final diagnosis was ypStage IIC (ypT4bypN0M0). All three cases underwent capecitabine-based adjuvant chemotherapy after radical surgery and patients are alive without recurrence.

**Conclusion:**

Neoadjuvant chemotherapy with CAPOX with or without bevacizumab followed by radical surgery could be an effective treatment option for LACC invading the urinary bladder.

## 1. Introduction

Colon cancer is the third most common cancer and one of the leading causes of cancer-associated mortality worldwide [1, 2]. Locally advanced colon cancer (LACC) is defined as a primary colon cancer invading adjacent organs or involving extensive regional lymph nodes in patients with Union for International Cancer Control stage IIC or III [3, 4]. Clinical guidelines recommend adjuvant chemotherapy as an established treatment for patients with stages IIC and III colorectal cancer [5]. The current standard of treatment for LACC is complete surgical removal of the tumor followed by adjuvant chemotherapy; however, the 5-year overall survival for LACC patients ranges from 37.3% in stage IIC patients to 28% in stage IIIC [6]. Thus, the oncological outcome of those patients remains unsatisfactory.

Neoadjuvant treatment before surgery has demonstrated oncological benefits in locally advanced rectal cancer [7, 8]. In contrast, the efficacy of neoadjuvant chemotherapy for LACC patients has been reported in limited case reports [9, 10]. Moreover, several studies have evaluated the feasibility and advantage of neoadjuvant treatment before surgery for LACC [3, 11–13]. Arredondo et al. investigated the midterm prognosis of LACC patients who underwent neoadjuvant chemotherapy followed by radical surgery, showing improved prognosis with a 5-year relapse-free survival of 85.6% and 5-year overall survival of 95.3% after the median follow-up of 40.1 months [13]. Furthermore, in terms of organ preservation, preoperative chemoradiotherapy for LACC involving adjacent organs could avoid extensive multivisceral resection and achieve an improved quality of life, especially in urinary function [14, 15]. However, the appropriate regimen for neoadjuvant chemotherapy for LACC patients has not been well-established.

We herein report three cases of locally advanced sigmoid colon cancer invading the urinary bladder in which neoadjuvant chemotherapy with capecitabine plus oxaliplatin (CAPOX) followed by surgery was effective in both improving oncological outcome and organ preservation.

## 2. Case Presentation

### 2.1. Case 1

**Figure 1:**
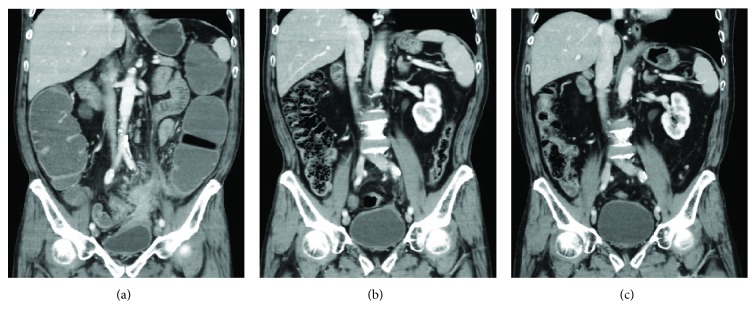
Coronal section of CT indicated the efficacy of neoadjuvant chemotherapy. Before chemotherapy (a). After 3 courses of CAPOX (b). After 5 courses, the tumor had disappeared (c).

A 78-year-old man presented with constipation and abdominal distension. His medical history was remarkable for diabetes mellitus and dilated cardiomyopathy. Laboratory data were unremarkable except for a slightly increased level of cancer antigen 19-9 (45.8 U/ml). Colonoscopy revealed a circumferential impassable tumor located 28 cm from the anal verge. Contrast-enhanced computed tomography (CT) showed irregular colonic wall thickening with infiltration into the urinary bladder (Figure 1(a)). No lymph node enlargement or distant metastases were found. Histological examination of the biopsy revealed adenocarcinoma. The RAS/RAF mutational status was not investigated. The pretreatment diagnosis was LACC stage IIC (T4bN0M0). We surmised that immediate radical surgery would result in substantial bladder resection with impaired urinary function. Therefore, neoadjuvant chemotherapy before radical surgery was planned.

Firstly, we performed loop transverse colostomy for symptomatic colonic obstruction. Secondly, we planned 6 courses of chemotherapy with CAPOX and the treatment was initiated 1 month after the loop colostomy. We did not add molecular targeted agents because of his reduced cardiac function (ejection fraction of 21%). Follow-up CT after 3 courses of CAPOX revealed significant tumor shrinkage (Figure 1(b)). However, after 5 courses of CAPOX, grade 1 neurotoxicity and grade 2 neutropenia were observed. As he did not wish to receive oxaliplatin, the 6th course comprised capecitabine alone. Follow-up CT after the treatment (5 courses of CAPOX and 1 course of capecitabine alone) showed tumor disappearance (Figure 1(c)).

**Figure 2:**
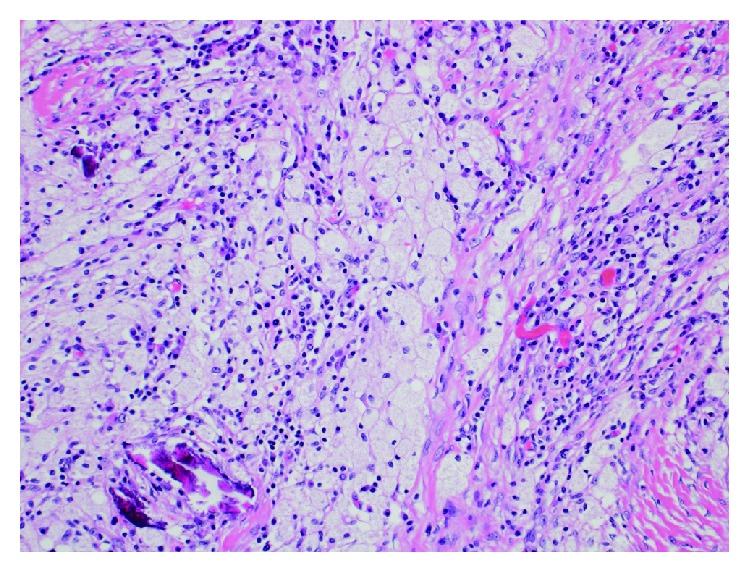
Pathological findings revealed no viable cells with collagen fibers. Original magnification: ×200.

Thereafter, we performed sigmoid colectomy, partial resection of the bladder, and diverting ileostomy. The postoperative course was uneventful, and he did not suffer from neurogenic voiding dysfunction or urinary incontinence. Well-tolerated pathological examination revealed no residual tumor cells in the resected specimen, which was consistent with pathological complete response (pCR) and grade 3 effect according to the Japanese Classification of Colorectal Carcinoma (Figure 2) [16]. The final pathological diagnosis was ypT0, ypN0, M0, ypStage 0. We then planned 8 courses of adjuvant chemotherapy with CAPOX. After 4 courses, he developed grade 2 neutropenia despite a dose reduction (70%) for oxaliplatin. Therefore, the following 4 courses of chemotherapy comprised capecitabine alone. Subsequently, he underwent ileostomy closure and had no recurrence at 25 months after the initial diagnosis.

### 2.2. Case 2

**Figure 3:**
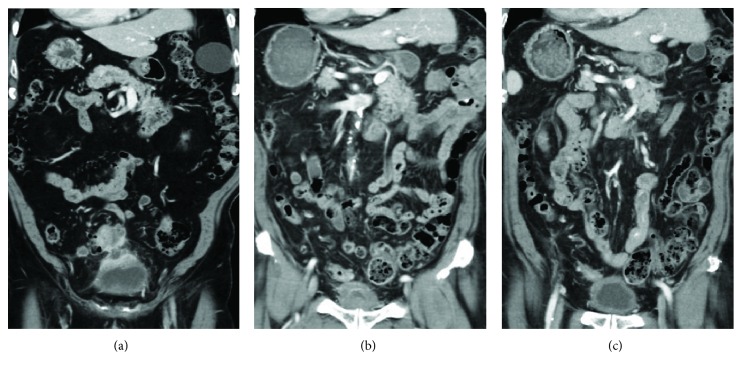
Before treatment, CT scan revealed a bulky mass in the sigmoid colon invading the bladder (a). After 6 courses of CAPOX (b). CT failed to detect the tumor after 13 courses of CAPOX plus bevacizumab (c).

A 79-year-old man presented with abdominal pain. His medical history was significant for high blood pressure without the need for medication. Laboratory data revealed an increased level of carcinoembryonic antigen (77.5 ng/ml) and a normal level of cancer antigen 19-9 (33.7 U/ml). Colonoscopy revealed an advanced tumor with 80% of the circumference in the sigmoid colon. Histological examination of the biopsy revealed adenocarcinoma. The RAS/RAF mutational status was not investigated. Contrast-enhanced CT showed irregular colonic wall thickening with infiltration into the urinary bladder (Figure 3(a)). There were enlarged regional lymph nodes suggestive of metastases but no distant metastasis. The pretreatment diagnosis was LACC stage IIIC (T4bN2M0).

Similar to Case 1, we initially performed loop transverse colostomy. Subsequently, we planned 6 courses of neoadjuvant chemotherapy with CAPOX plus bevacizumab followed by surgery; however, the patient wished to continue chemotherapy rather than have surgery. No significant adverse events occurred. After 13 courses, CT showed significant tumor shrinkage and reduction of bladder wall thickening (Figure 3(c)). Colonoscopy revealed the disappearance of the primary tumor. Thereafter, he agreed to undergo surgery.

**Figure 4:**
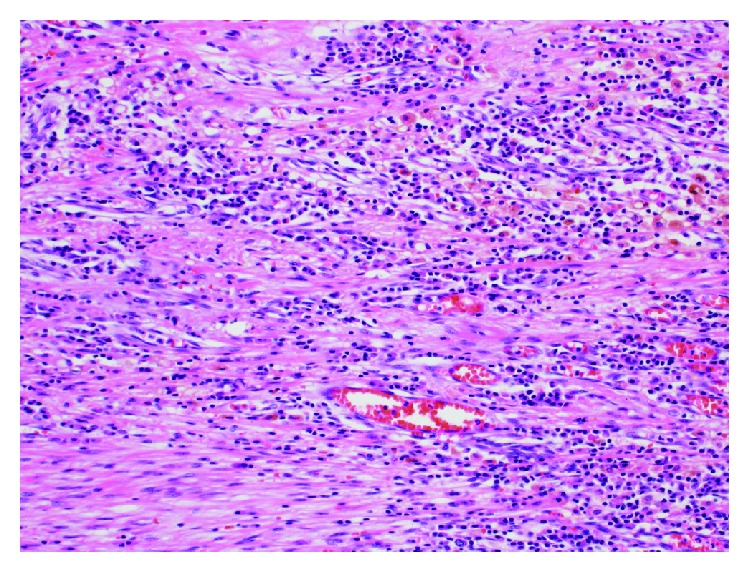
Microscopic findings showed no residual tumor cells with foci of fibrotic tissue and inflammatory cell infiltration. Original magnification: ×200.

We performed Hartmann's operation with partial resection of the bladder. The postoperative course was uneventful, and he did not suffer from neurogenic voiding dysfunction or urinary incontinence. Pathological examination revealed no residual tumor cells in the resected specimen with foci of fibrotic tissue and inflammatory cell infiltration, which was consistent with pCR and grade 3 effect according to the Japanese Classification of Colorectal Carcinoma (Figure 4) [16]. The final pathological diagnosis was ypT0, ypN0, M0, ypStage 0. He then received 8 courses of adjuvant chemotherapy with CAPOX. Hartmann's reversal was not performed because he did not wish to do so. There was no recurrence at 65 months after the initial diagnosis.

### 2.3. Case 3

**Figure 5:**
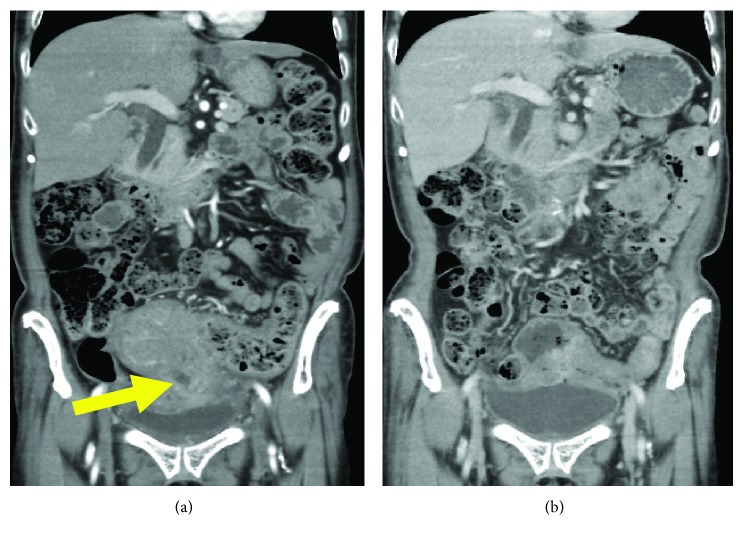
Contrast-enhanced CT showed an irregular mass with infiltration into the bladder. The arrow indicates the communication between the mass and the bladder (a). After 5 courses of neoadjuvant chemotherapy, CT scan revealed shrinkage of the primary tumor (b).

A 74-year-old woman presented with body weight loss. Her medical history included hypertension and diabetes mellitus, both of which were well-controlled by medication. Laboratory data were unremarkable except for decreased hemoglobin (9.1 g/dl). Tumor markers were within normal limits. Colonoscopy revealed a circumferential tumor in the sigmoid colon. Histological examination of the biopsy revealed RAS-mutant adenocarcinoma. The RAF mutational status was not investigated. Contrast-enhanced CT showed irregular colonic wall thickening with massive involvement of the urinary bladder (Figure 5(a)). There were enlarged regional lymph nodes suggestive of metastases but no distant metastasis. The pretreatment diagnosis was LACC stage IIIC (T4bN2M0).

Similar to Cases 1 and 2, we initially performed loop transverse colostomy. Subsequently, we planned 6 courses of chemotherapy with CAPOX plus bevacizumab. The patient eventually received 5 courses of CAPOX plus bevacizumab and the remaining 1 course without oxaliplatin due to grade 2 neurotoxicity. Follow-up CT after the neoadjuvant chemotherapy revealed significant tumor shrinkage (Figure 5(b)).

**Figure 6:**
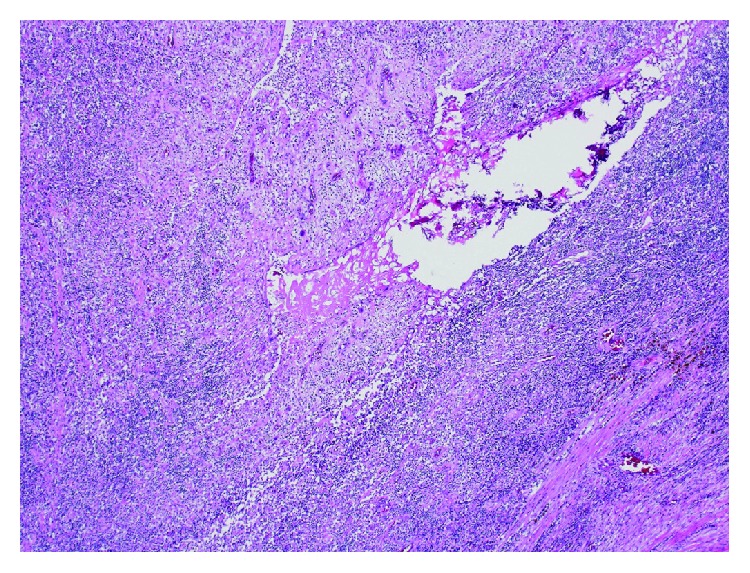
Viable cells were observed in less than one third of the lesion. The histological effect was consistent with grade 1a. Original magnification: ×40.

Thereafter, we performed sigmoid colectomy and partial resection of the bladder. The postoperative course was uneventful, and the patient did not suffer from neurogenic voiding dysfunction or urinary incontinence. Pathological examination revealed that tumor cells or degeneration was present in less than one third of the entire lesion, which was consistent with grade 1a effect according to the Japanese Classification of Colorectal Carcinoma (Figure 6) [16]. The final diagnosis was ypT4b, ypN0, M0, ypStage IIC. She subsequently received 8 courses of adjuvant chemotherapy with capecitabine alone. There was no recurrence at 16 months after the initial diagnosis.

## 3. Discussion

The efficacy of neoadjuvant chemotherapy for potentially resectable LACC is uncertain. En bloc multivisceral resection is the standard surgical procedure for LACC invading adjacent structures [17, 18]. Although R0 resection rates ranged from 65.0% to 93.1%, postoperative rates of complications (25.8-33.0%) and mortality (6.9-7.5%) were high. In addition, aggressive surgical procedures may damage the function of adjacent organs when resected together. A previous study indicated that the urinary bladder is the most frequently involved organ in patients with LACC [19]. Therefore, oncologic clearance and organ preservation must be carefully balanced. Recently, several studies have shown the efficacy of neoadjuvant treatment followed by surgery for LACC [3, 11–13]. In this report, we suggested a new treatment strategy of neoadjuvant chemotherapy using CAPOX with or without bevacizumab to improve cancer prognosis and preserve urinary function for patients with T4b LACC involving the urinary bladder.

In our cases, CT scan showed substantial cancer invasion into the urinary bladder. At the pretreatment discussions, extensive resection of the bladder was deemed necessary in Cases 1 and 2, and subtotal or total cystectomy might be appropriate for curative resection in Case 3. The invasion to the urinary bladder prompted the administration of neoadjuvant chemotherapy, and this strategy successfully led to tumor shrinkage in all cases. The operative findings of Cases 1 and 2 showed spreading inflammation around the sigmoid colon, where the primary tumor was originally located. However, intraoperative discrimination of inflammatory adhesions from malignant invasion is extremely difficult; therefore, we performed combined resection of the affected area of the bladder including the detrusor muscle layer. On the other hand, in Case 3, CT scan after neoadjuvant chemotherapy still indicated the presence of communication between the sigmoid colon and the bladder. Therefore, we performed sigmoid colectomy with full-thickness resection (including mucosa) of the bladder. After successful en bloc resection of the primary lesion, the defect of the bladder was then closed in an interrupted layer-to-layer manner. Although partial cystectomy was necessary in all cases, they did not complain of any related symptoms such as pollakiuria, urinary urgency, urinary incontinence, and incomplete voiding after surgery. Thus, urinary function was well preserved in all cases. We assumed that our treatment strategy was effective in organ preservation.

With respect to the oncological outcome, Arredondo et al. reported good midterm prognosis for LACC patients with neoadjuvant chemotherapy followed by radical surgery [13]. They concluded that cancer survival of those patients was encouraging. A systemic review and meta-analysis demonstrated that patients with locally advanced rectal cancer who had complete response to neoadjuvant chemoradiotherapy exhibited excellent long-term survival and low local recurrence rates [20]. Another study indicated that locally advanced rectal cancer patients with pCR after chemoradiotherapy achieved improved disease-free survival and overall survival rates than those without pCR [21]. Currently, a phase II trial is ongoing which evaluates disease-free survival in patients with locally advanced rectal cancer treated with chemoradiation plus induction or consolidation chemotherapy [22]. The results of this trial would certainly bring new insights to the field of neoadjuvant treatment for locally advanced rectal cancer.

In contrast, there are few studies investigating the survival rates of LACC patients with pCR. Nevertheless, it is conceivable that LACC patients with pCR might have improved prognosis and survival rates, similar to rectal cancer patients. In fact, in Case 2 of our report, the patient showed 61 months of recurrence-free survival after the initial treatment. This result supports the concept that LACC patients who achieved pCR after neoadjuvant chemotherapy followed by radical surgery can survive longer than patients without neoadjuvant chemotherapy.

One of the concerns with neoadjuvant chemotherapy for LACC is the choice of regimen. Pilot clinical trials adopted oxaliplatin, fluorouracil, and leucovorin (OxMdG) with or without panitumumab, CAPOX with or without panitumumab, and infusional fluorouracil, leucovorin, and oxaliplatin (FOLFOX) without molecular agent [3, 11, 13]. To date, the standard neoadjuvant regimen for LACC patients has not been established. FOLFOX and CAPOX are the standard adjuvant chemotherapy regimens for high-risk stage II and III colorectal cancer patients and are also used as first-line chemotherapy for metastatic colorectal cancer (mCRC) [23, 24]. A phase III clinical trial is recently in progress to compare the efficacy and safety of the neoadjuvant and adjuvant CAPOX chemotherapy with the postoperative one for locally advanced resectable colon cancer [25]. Furthermore, CAPOX can be used as radiosensitizer for neoadjuvant chemoradiation in downstaging locally advanced rectal cancer [26]. We selected CAPOX as the neoadjuvant chemotherapy because we believed it was advantageous that the medications could be delivered without the use of a venous reservoir implant. Moreover, the addition of bevacizumab to an oxaliplatin-based regimen is the standard first-line chemotherapy for mCRC cancer patients; we thus added bevacizumab to the neoadjuvant chemotherapy regimen unless contraindicated [27]. In Case 1, the ejection fraction was 21% as determined by echocardiography; hence, we did not add bevacizumab to his neoadjuvant treatment. In this report, all three patients were elderly (in their 70s) with some comorbidities. Younger patients with fewer comorbidities could have benefited from other triplet regimens such as fluorouracil, leucovorin, oxaliplatin, and irinotecan (FOLFOXIRI) plus bevacizumab [28].

Recent reports suggested that primary tumor location in terms of right- versus left-sided cancer is associated with survival difference in mCRC patients [29–31]. Tejpar et al. investigated the prognostic relevance of primary tumor location in patients with RAS wild-type mCRC [32] and concluded that patients with left-sided mCRC have remarkably better prognosis than patients with right-sided mCRC. Moreover, they indicated that first-line infusional fluorouracil, leucovorin, and irinotecan (FOLFIRI) plus cetuximab benefitted patients with left-sided cancer. Therefore, RAS testing is strongly recommended before the induction of neoadjuvant chemotherapy for LACC, for the choice of antiepidermal growth factor receptor (EGFR) agents in the treatment regimen. In our cases, the RAS mutation was positive in Case 3; however, it was not investigated in Cases 1 and 2. Currently, the RAS/RAF testing is universally available for LACC in our institution and the status of which would determine our therapeutic approach.

Another therapeutic option for obstructive sigmoid colon cancer is the self-expanding metallic stent (SEMS), which has been widely applied in patients with malignant large bowel obstruction [33]. The use of a SEMS as a bridge to surgery is advantageous as it avoids colostomy. However, as Lee et al. implied that chemotherapy after deploying a SEMS in mCRC patients might cause intestinal perforation, we opted for colostomy creation followed by neoadjuvant chemotherapy for safer administration during continuous chemotherapy [34]. Moreover, guidelines indicated that SEMS placement is not recommended in patients treated with antiangiogenic agents [35]. The optimal timing of radical surgery after neoadjuvant chemotherapy has never been investigated in LACC. Due to the potential outgrowth of the primary tumor during neoadjuvant chemotherapy, we might miss the appropriate timing for surgical intervention. Further prospective studies are required to evaluate the feasibility of this strategy.

Although neoadjuvant chemotherapy regimens for LACC patients remain unknown, it is important to select the appropriate regimen depending on primary tumor location, RAS status, and comorbidity of the patients.

## 4. Conclusion

Neoadjuvant chemotherapy with CAPOX with or without bevacizumab followed by radical surgery could be an effective treatment option in terms of both improving oncological outcome and organ preservation for LACC invading the urinary bladder. Further prospective studies are needed to evaluate the feasibility of this treatment strategy.

## References

[1] Torre L. A., Bray F., Siegel R. L., Ferlay J., Lortet-Tieulent J., Jemal A. (2015). Global cancer statistics, 2012. *CA: A Cancer Journal for Clinicians*.

[2] Siegel R. L., Miller K. D., Jemal A. (2018). Cancer statistics, 2018. *CA: A Cancer Journal for Clinicians*.

[3] FOxTROT Collaborative Group (2012). Feasibility of preoperative chemotherapy for locally advanced, operable colon cancer: the pilot phase of a randomised controlled trial. *The Lancet Oncology*.

[4] Smith N. J., Bees N., Barbachano Y., Norman A. R., Swift R. I., Brown G. (2007). Preoperative computed tomography staging of nonmetastatic colon cancer predicts outcome: implications for clinical trials. *British Journal of Cancer*.

[5] Benson A. B., Schrag D., Somerfield M. R. (2004). American Society of Clinical Oncology recommendations on adjuvant chemotherapy for stage II colon cancer. *Journal of Clinical Oncology*.

[6] Edge S., Byrd D. R., Compton C. C., Fritz A. G., Greene F., Trotti A. (2010). *AJCC Cancer Staging Manual*.

[7] Sauer R., Becker H., Hohenberger W. (2004). Preoperative versus postoperative chemoradiotherapy for rectal cancer. *The New England Journal of Medicine*.

[8] Roh M. S., Colangelo L. H., O'Connell M. J. (2009). Preoperative multimodality therapy improves disease-free survival in patients with carcinoma of the rectum: NSABP R-03. *Journal of Clinical Oncology*.

[9] Yoshitomi M., Hashida H., Nomura A., Ueda S., Terajima H., Osaki N. (2014). *Gan To Kagaku Ryoho*.

[10] Tomizawa K., Miura Y., Fukui Y. (2017). Curative resection for locally advanced sigmoid colon cancer using neoadjuvant chemotherapy with FOLFOX plus panitumumab: a case report. *International Journal of Surgery Case Reports*.

[11] Jakobsen A., Andersen F., Fischer A. (2015). Neoadjuvant chemotherapy in locally advanced colon cancer. A phase II trial. *Acta Oncologica*.

[12] Huang C.-M., Huang M.-Y., Ma C.-J. (2017). Neoadjuvant FOLFOX chemotherapy combined with radiotherapy followed by radical resection in patients with locally advanced colon cancer. *Radiation Oncology*.

[13] Arredondo J., Baixauli J., Pastor C. (2017). Mid-term oncologic outcome of a novel approach for locally advanced colon cancer with neoadjuvant chemotherapy and surgery. *Clinical and Translational Oncology*.

[14] Qiu B., Ding P. R., Cai L. (2016). Outcomes of preoperative chemoradiotherapy followed by surgery in patients with unresectable locally advanced sigmoid colon cancer. *Chinese Journal of Cancer*.

[15] Chang H., Yu X., Xiao W. W. (2018). Neoadjuvant chemoradiotherapy followed by surgery in patients with unresectable locally advanced colon cancer: a prospective observational study. *OncoTargets and Therapy*.

[16] Japanese Society of Cancer of the Colon and Rectum (2018). *Japanese Classification of the Colorectal, Appendiceal, and Anal Carcinoma*.

[17] Lehnert T., Methner M., Pollok A., Schaible A., Hinz U., Herfarth C. (2002). Multivisceral resection for locally advanced primary colon and rectal cancer: an analysis of prognostic factors in 201 patients. *Annals of Surgery*.

[18] Croner R. S., Merkel S., Papadopoulos T., Schellerer V., Hohenberger W., Goehl J. (2009). Multivisceral resection for colon carcinoma. *Diseases of the Colon and Rectum*.

[19] Talamonti M. S., Shumate C. R., Carlson G. W., Curley S. A. (1993). Locally advanced carcinoma of the colon and rectum involving the urinary bladder. *Surgery, Gynecology & Obstetrics*.

[20] Martin S. T., Heneghan H. M., Winter D. C. (2012). Systematic review and meta-analysis of outcomes following pathological complete response to neoadjuvant chemoradiotherapy for rectal cancer. *British Journal of Surgery*.

[21] Maas M., Nelemans P. J., Valentini V. (2010). Long-term outcome in patients with a pathological complete response after chemoradiation for rectal cancer: a pooled analysis of individual patient data. *The Lancet Oncology*.

[22] Aguilar J. G. Trial evaluating 3-year disease free survival in patients with locally advanced rectal cancer treated with chemoradiation plus induction or consolidation chemotherapy and total mesorectal excision or non-operative management. https://clinicaltrials.gov/ct2/show/NCT02008656.

[23] Cassidy J., Clarke S., Díaz-Rubio E. (2008). Randomized phase III study of capecitabine plus oxaliplatin compared with fluorouracil/folinic acid plus oxaliplatin as first-line therapy for metastatic colorectal cancer. *Journal of Clinical Oncology*.

[24] André T., de Gramont A., Vernerey D. (2015). Adjuvant fluorouracil, leucovorin, and oxaliplatin in stage II to III colon cancer: updated 10-year survival and outcomes according to *BRAF* mutation and mismatch repair status of the MOSAIC study. *Journal of Clinical Oncology*.

[25] Liu F., Tong T., Huang D. (2019). CapeOX perioperative chemotherapy versus postoperative chemotherapy for locally advanced resectable colon cancer: protocol for a two-period randomised controlled phase III trial. *BMJ Open*.

[26] Saha A., Ghosh S., Roy C., Saha M., Choudhury K., Chatterjee K. (2015). A randomized controlled pilot study to compare capecitabine-oxaliplatin with 5-FU-leucovorin as neoadjuvant concurrent chemoradiation in locally advanced adenocarcinoma of rectum. *Journal of Cancer Research and Therapeutics*.

[27] Saltz L. B., Clarke S., Díaz-Rubio E. (2008). Bevacizumab in combination with oxaliplatin-based chemotherapy as first-line therapy in metastatic colorectal cancer: a randomized phase III study. *Journal of Clinical Oncology*.

[28] Loupakis F., Cremolini C., Masi G. (2014). Initial therapy with FOLFOXIRI and bevacizumab for metastatic colorectal cancer. *The New England Journal of Medicine*.

[29] Price T. J., Beeke C., Ullah S. (2015). Does the primary site of colorectal cancer impact outcomes for patients with metastatic disease?. *Cancer*.

[30] Brulé S. Y., Jonker D. J., Karapetis C. S. (2015). Location of colon cancer (right-sided versus left-sided) as a prognostic factor and a predictor of benefit from cetuximab in NCIC CO.17. *European Journal of Cancer*.

[31] Engstrand J., Nilsson H., Stromberg C., Jonas E., Freedman J. (2018). Colorectal cancer liver metastases - a population-based study on incidence, management and survival. *BMC Cancer*.

[32] Tejpar S., Stintzing S., Ciardiello F. (2017). Prognostic and Predictive Relevance of Primary Tumor Location in Patients With *RAS* Wild-Type Metastatic Colorectal Cancer: Retrospective Analyses of the CRYSTAL and FIRE-3 Trials. *JAMA Oncology*.

[33] Zhang Y., Shi J., Shi B., Song C. Y., Xie W. F., Chen Y. X. (2012). Self-expanding metallic stent as a bridge to surgery versus emergency surgery for obstructive colorectal cancer: a meta-analysis. *Surgical Endoscopy*.

[34] Lee W. S., Baek J. H., Kang J. M., Choi S., Kwon K. A. (2012). The outcome after stent placement or surgery as the initial treatment for obstructive primary tumor in patients with stage IV colon cancer. *American Journal of Surgery*.

[35] van Hooft J., van Halsema E., Vanbiervliet G. (2014). Self-expandable metal stents for obstructing colonic and extracolonic cancer: European Society of Gastrointestinal Endoscopy (ESGE) Clinical Guideline. *Endoscopy*.

